# Case Report: Primary solid pseudopapillary neoplasm of the ovary with “cholesteroma-like” denaturation

**DOI:** 10.3389/fonc.2025.1514460

**Published:** 2025-03-06

**Authors:** Ziqing Zhao, Jiahui Lin, Tingting Bai, Hongfeng Liao, Zhengjin Liu

**Affiliations:** ^1^ Department of Pathology, Zhongshan Hospital of Xiamen University, School of Medicine, Xiamen University, Xiamen, China; ^2^ Department of Radiology, Zhongshan Hospital of Xiamen University, School of Medicine, Xiamen University, Xiamen, China

**Keywords:** solid pseudopapillary neoplasm, ovary, β-catenin, primarily, “cholesteroma-like” denaturation

## Abstract

Solid pseudopapillary neoplasms (SPNs) primarily arise in the pancreas and are uncommon in the ovaries. Here, we present a case of ovarian-origin SPN. Alongside the typical solid and pseudopapillary structures, “cholesteroma-like” denaturation areas and tissue degeneration regions are also observed. Immunohistochemistry analysis demonstrates positive results for β-catenin (nucleus), CD99 (dot-like), CD56, and vimentin. Imaging studies rule out pancreatic or other origins. This study aims to enhance comprehension, diagnosis, and differential diagnosis of primary ovarian SPN among pathologists and clinicians, as well as to investigate the origin and management of primary solid pseudopapillary tumors in the ovary.

## Introduction

Solid pseudopapillary neoplasms (SPNs) are uncommon low-grade malignant neoplasms primarily found in the pancreas, representing 0.3-2.7% of all exocrine pancreatic neoplasms (1). The term “solid pseudopapillary neoplasm (or neoplasm)” was officially adopted by the World Health Organization (WHO) in 1996, following initial characterization by V.K. Frantz in 1959 (2). Although previously identified under different names, the consistent nomenclature was established by WHO. This condition typically affects young women, with initial symptoms often nonspecific, such as abdominal pain or discomfort (1). Imaging typically reveals irregular cystic solid masses. The differentiation pathway of SPNs remains uncertain, with molecular studies indicating frequent β-catenin gene mutations in solid pseudopapillary tumors.

In recent years, an increasing number of reports have highlighted the occurrence of SPNs beyond the pancreas, including locations such as the retroperitoneum, omentum, liver, gastroduodenum, and paratestis. While SPNs in the ovaries have been rarely reported, recent findings indicate the ovaries as a primary site for SPNs outside the pancreas (3). The case presented in this study involves an ovarian SPN, exhibiting clinical, imaging, histological, immunohistochemical, and molecular pathological features consistent with previously documented cases, along with the identification of unique morphological characteristics.

## Case presentation

**Figure 1:**
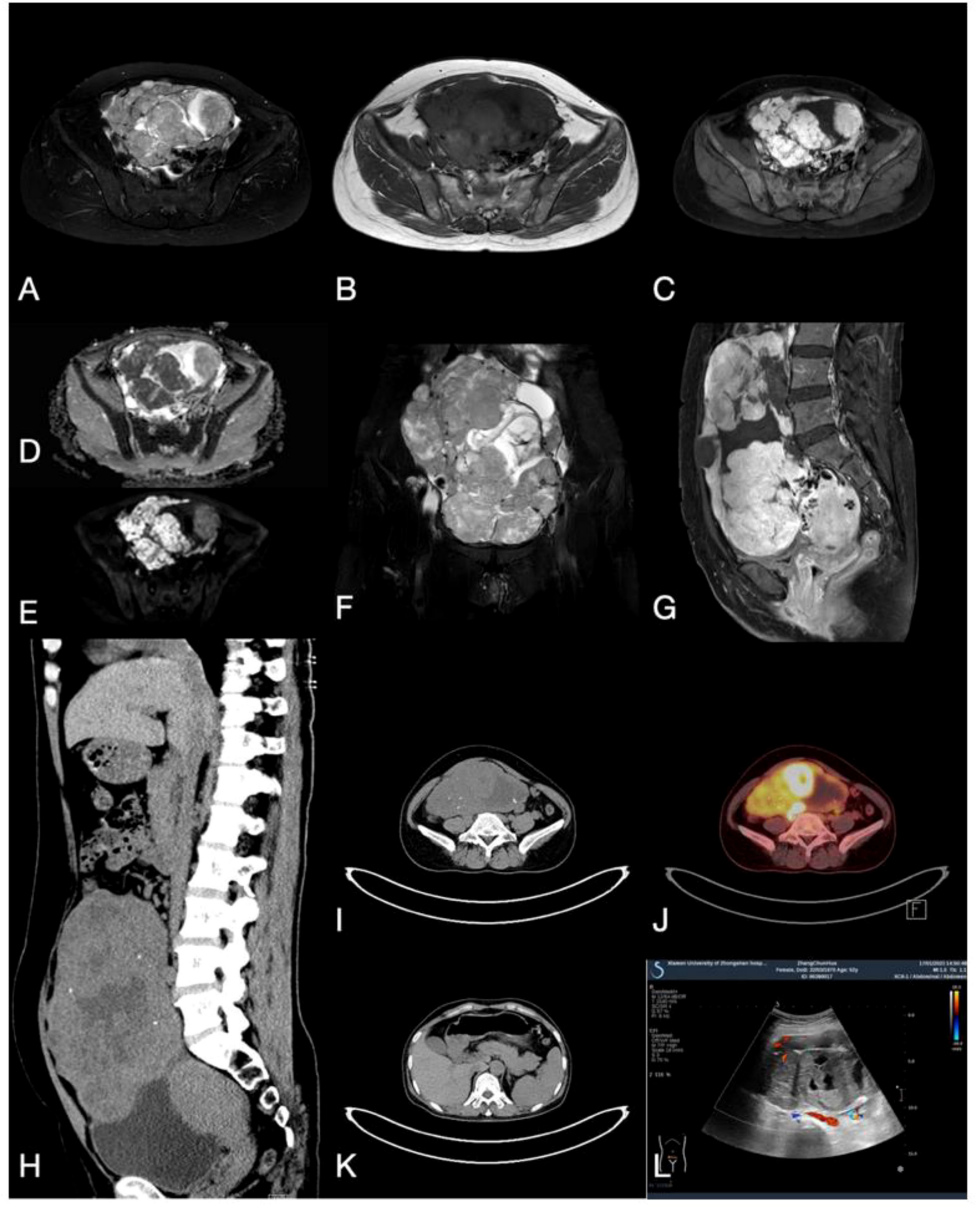
**(A, B)** MRI T2 and Tl weighted imaging revealed a cystic solid mass with mixed signals within the ovary. **(C)** MRI enhanced transverse imaging. **(D, E)** Diffusion-weighted MRI, ADC map. **(F)** Coronal T2 weighted imaging. **(G)** MRI enhanced sagittal imaging. **(H, I)** CT scan demonstrates a cystic-solid mass with mixed density and scattered calcification in the sagittal and transverse planes. **(J)** PET-CT indicates high uptake in the solid tumor component. **(K)** CT plain scan revealed no lesions in the pancreas, liver, or other regions. **(L)** Ultrasound indicates the presence of a cystic and solid mass with mixed echoes within the ovary.

A 52-year-old female presented with paroxysmal lower abdominal pain. The patient’s medical history includes hypertension and a right renal cyst. There is no history of genetic, metabolic disorders among family members, nor infectious diseases in the patient. Computed Tomography (CT) and Magnetic Resonance Imaging (MRI) indicated a large heterogeneous mass in the abdominal and pelvic cavity, characterized by solid components, cystic alterations, and calcifications. The mass demonstrates clear demarcation, leading to displacement of adjacent intestinal tracts without evident erosion or infiltration. The greater omentum appears uninvolved, with the lesion measuring approximately 172.1 x 95.3 mm in its largest dimensions (**Figures 1A–I**). Positron emission tomography-computed tomography (PET-CT) revealed increased uptake in the solid tumor mass (**Figure 1J**). Additionally, CT imaging showed no remarkable abnormalities in the pancreas or other organs (**Figure 1K**). Ultrasound (US) examination revealed a sizable solid ovarian mass (**Figure 1L**). Laboratory analysis indicated elevated CA125 levels at 43.54U/mL (normal range: 0.00-35.00 U/mL).

**Figure 2:**
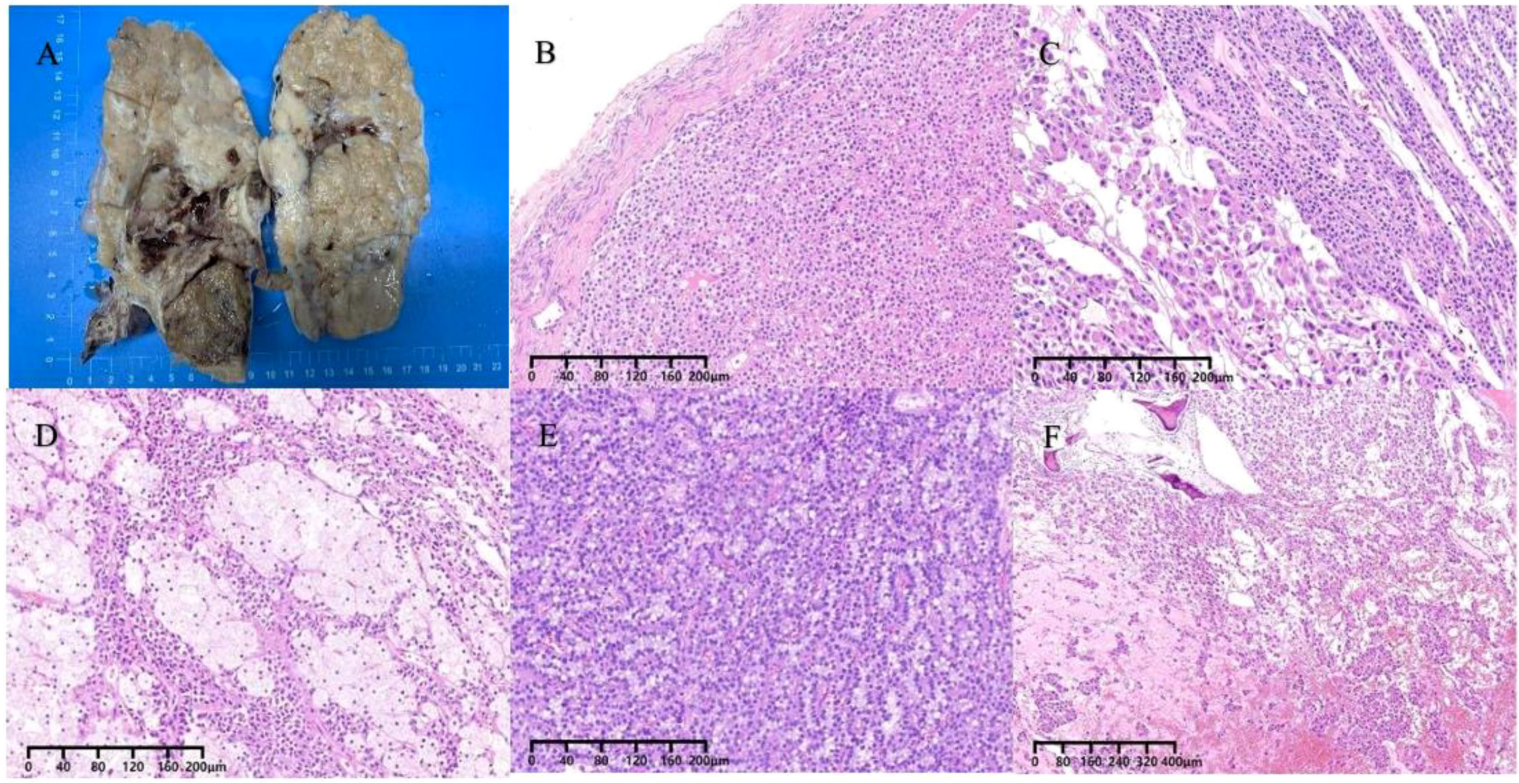
**(A)** The gross cross-section of the mass appears cystic and solid. **(B)** The tumor is situated within the ovarian parenchyma, with indistinct margins and the presence of residual normal ovarian cortex. **(C, D)** Shifting transitions are visible between different regions. **(E)** Vacuoles within the cytoplasm. **(F)** Necrosis with hemorrhage, focal calcified areas.

**Figure 3:**
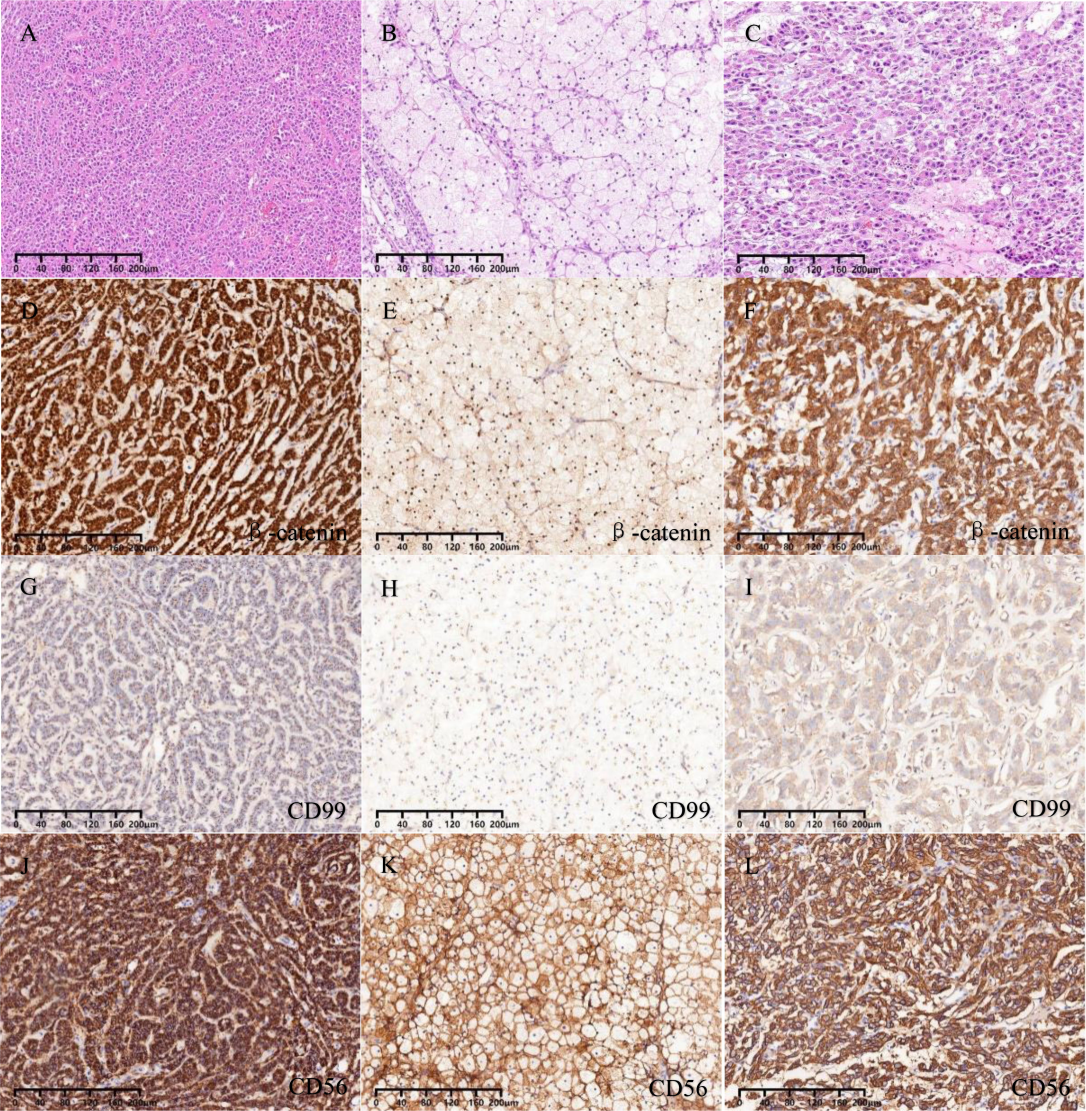
**(A)** Solid, trabecular, and pseudopapillary areas. **(B)** ”cholesteroma-like” formations areas. **(C)** Tissue regions of degeneration. **(D-F)** Nuclear and cytoplasmic staining for β-catenin. **(G-I)** Dot-like positive staining for CD99. **(J-L)** Membranous staining for CD56.

Subsequently, the patient underwent oophorectomy. The excised specimen appeared as a grayish-red irregularly shaped tissue block, measuring 18.5 x 14.3 x 9.8 cm, with a surface capsule thickness ranging from approximately 0.3 to 0.1 cm. Multiple solid protrusions covered the tissue surface, comprising both cystic and solid regions. The solid region displayed a grayish-yellow coloration, with focal areas of grayish-white and dark yellow, exhibiting a solid and medium texture. Within the cystic area, multiple cystic cavities were observed, with the largest measuring 7.8 x 3.2 cm in diameter. The inner cyst wall appeared smooth, with visible protrusions. Dark red and dark yellow gel-like substances were found within the cystic cavities (**Figure 2A**). The patient’s uterus, left fallopian tube, and right adnexa demonstrated normal morphology. Histologically, the neoplasm was observed within the ovarian parenchyma with indistinct boundaries. Normal ovarian cortical remnants were identified (**Figure 2B**). The neoplasm exhibited a complex architecture, comprising solid, trabecular, glandular, and occasionally pseudopapillary structures (**Figure 3A**). Additionally, some areas exhibited features reminiscent of “cholesteroma-like” formations (**Figure 3B**), while others displayed tissue degeneration characterized by abundant eosinophilic cytoplasm, enlarged nuclei, and prominent nucleoli (**Figure 3C**). Notably, distinct transitions between different regions were evident in the specimen (**Figures 2C, D**). In addition, intracellular vacuoles were noted in certain areas, displaying a clear appearance (**Figure 2E**). The nuclei of the neoplasm appeared round or oval, characterized by fine chromatin and occasional nuclear grooves, with no evident nuclear division observed. The interstitium exhibited a dense network of capillaries. Local areas of necrosis, accompanied by hemorrhage and focal calcification, were identified (**Figure 2F**). Subsequent sampling revealed the absence of ectopic pancreatic tissue or teratoma components within the specimen.

**Figure 4:**
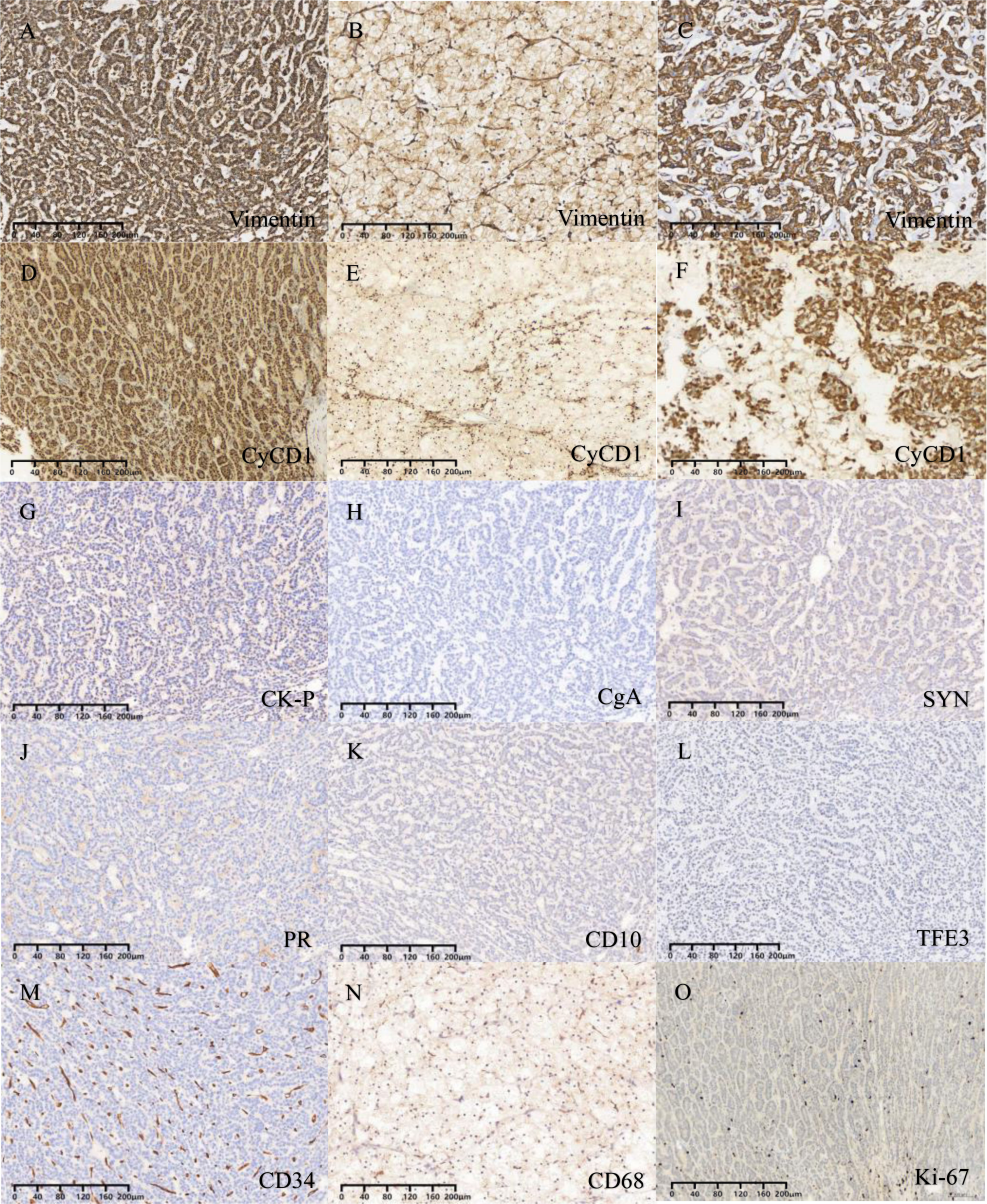
**(A-C)** Vimentin is expressed in all three different regions. **(D-F)** CyCD1 is expressed in all three different regions. **(G)** Focal expression of CK-P. **(H-N)** Negative staining for CgA, SYN, PR, CD10, TFE3, CD34 and CD68. **(O)** The Ki-67 proliferation index was approximately 3%.

The immunohistochemical staining outcomes revealed consistent expression patterns across three distinct regions (**Figures 3D–L**, **4A–F**). The neoplastic cells exhibited positivity for β-catenin (nuclear), CD99 (dot-like), CD56, Vimentin, CD10, SYN, and cytokeratin (focal weak), while E-Cad, TFE3, CD34, chromaffin, inhibin, and progesterone receptors displayed negative staining. The Ki-67 proliferation index was estimated to be around 3%. Notably, in contrast to genuine pancreatic pseudopapillary neoplasms, the “cholesteroma-like” formations of the neoplasm demonstrated a lack of CD68 (KP-1) expression, confirming its non-histiocytic nature, a distinctive feature of our case (**Figures 4G–O**).

The patient has no recurrence or metastasis so far. Based on the above results, the patient was diagnosed with primary ovarian SPN.

## Discussion

Primary ovarian SPNs are exceedingly rare, with only 13 cases, inclusive of the present report, documented in the existing literature (4–13). The age of onset for ovarian SPNs ranges from 18 to 57 years, typically manifesting with abdominal swelling as the predominant symptom. Diagnosis is often established through ultrasound or CT imaging, revealing ovarian masses with tumor diameters ranging from 3 cm to 25.5 cm. We have comprehensively summarized the clinicopathological, histopathological, and immunohistochemical characteristics of these 13 cases of primary ovarian SPNs (**Tables 1**, **2**).

**Table 1:** Clinicopathologic and histopathological features of all reported cases of solid pseudopapillary neoplasms of the ovarian origin.

	This case	Kushner (2020) (13)	Singh (2018) (12)	Komforti (2018) (11)	Gahlot (2016) (10)	He (2015) (9)	Kominami (2014) (8)	Stoll (2012) (7)	Syriac (2012) (6)	Cheuk (2011) (5)	Deshpande (2010) (4)	Deshpande (2010) (4)	Deshpande (2010) (4)
Age (years)	52	40	49	18	25	39	18	48	45	25	17	57	21
Maximum diameter (cm)	7.8	20	3.8	17.5	12	6	9.5	8	7.5	16.5	25.5	3	14
General	Solid and cystic	Polycystic	Solid and cystic	Solid and cystic	Solid and cystic	Cystic	Solid and cystic	Polycystic	Solid and cystic	Solid and cystic	Solid and cystic	Solid and cystic	Solid and cystic
Boundaries	Clear	NA	Clear	Clear	NA	Unclear	Clear	NA	Unclear	NA	Clear	Clear	Clear
Histological structural	Beam-like, glandular, locally papillary, "cholesterol-like" patchy	Solid cell nest	Flaky, Nest-like, False Nipple	Solid and false nipples	False nipples	Flaky, nest-like, false nipple	Solid and false nipples	Flaky, nest-like, false nipple	Solid and false nipples	False nipples	Flaky, nest-like, false nipple	Solid, focal false nipple, microcapsules	False nipples
Cellular	Epithelial-like, round	Polygon	Polygon	Signet-style	Polygon	Polygon	Polygon	Epithelial-like and plasma cell-like	Round shape, with mild pleomorphism and atypia	Cube shape	Polygon	Polygon	Polygon
Cytoplasmic	Acidophilic, foam-like	Acidophilic	Acidophilic, granular, foam	Transparent, foam	Acidophilic	Lightly tinted, foam-like	Acidophilic and vacuolar	Acidophilic and vacuolar	Mild acidophilic	Acidophilic granular, transparent	Transparent and foam occasionally	Acidophilic, some cytoplasm transparent	foam-like
Acidophilic body	NO	NA	Yes	Yes	NA	Yes	Yes	Yes	NA	Yes	Yes	Yes	NA
Nucleus	Round or oval	Light dyeing	Round or oval	Round or elliptical	Round, elliptical, vesicular	Round or oval	Round or elliptical	Round or elliptical	NA	Round	NA	Round or oval	NA
Chromatin	Delicate	NA	Delicate	Delicate	NA	Fine particles	Delicate	Delicate	Disperse	Fine particles	Delicate	NA	NA
Nuclear sulcus	NA	NA	Occasionally	Yes	NA	Yes	NA	NA	NA	NA	Yes	Yes	Yes
Mitosis	Not easily visible	Rare	NO	NO	NO	Few	NO	Not evident	NA	NO	NO	NO	NA
Interstitial	Capillary vessel	Capillary vessel	Thick fibrous septa and a vascular network with thin-walled vessels	Thin fibrous septa and capillary network	NA	Vascular richness	NA	Focal perivascular matrix hyaline degeneration	Small blood vessels and fibrous septa	Fiber septum and vascular network	Strip-like fibrous septa and capillary networks wrapped in transparent bands	Fractured gap	Vessels
LVI	NO	Yes	NO	NO	NA	NA	NA	NA	Yes	NA	NO	NA	NA
Neurological invasion	NO	NA	NO	NA	NA	NA	NA	NA	NA	NA	NA	NA	NA
Other	Necrosis, bleeding, calcification, some tumor tissue undergoing degeneration	NA	NA	Degenerative changes and extensive ischemic necrosis	NA	NA	NA	NA	Extensive necrosis	Hemorrhagic lesion	Infarct	NA	Cystic cavity filled with glial tissue
Metastasis	NO	Yes	NA	NA	NA	NA	NA	NA	Yes	NA	NO	NA	NA
Follow-up	NRM to date	RM at 6 mo	NRM at 12 mo	NRM at 3 mo	NRM at 18 mo	R at 3 mo	NA	NRM at 9 mo	Death after 8 mo	NRM at 12 mo	NRM at 6 mo	R at 7 yr	NA

LVI, lymphovascular invasion; NA, not available; R, recurrence; RM, recurrence and metastasis; NRM, no recurrence or metastasis.

**Table 2:** Immunohistochemical features of all reported cases of solid pseudopapillary neoplasms of the ovarian origin. .

	This case	Kushner (2020) (13)	Singh (2018) (12)	Komforti (2018) (11)	Gahlot (2016) (10)	He (2015) (9)	Kominami (2014) (8)	Stoll (2012) (7)	Syriac (2012) (6)	Cheuk (2011) (5)	Deshpande (2010) (4)	Deshpande (2010) (4)	Deshpande (2010) (4)
β-Catenin	NC+	N+	N+	NC+	NC+	N+	NC+	N+	NC+	NC+	NC+	NC+	N+
CD99	DL+	NA	M+	NA	NA	D+	NA	NA	NA	NA	NA	NA	NA
CD56	M+	M+	M+	M+	M+	M+	M+	DW+	–	M+-	M+	M+	NA
Vimentin	NC+	C+	C+	F+	NA	NC+	NC+	NC+	NC+	NA	–	NA	NA
CD10	F+	NA	P+	P+	MC+	NA	P+	P+	–	MC+	F+	NA	NA
SYN	F+	F+	C+	NA	NA	–	FW+	–	–	FW+	W+	FW+	NA
CgA	–	–	–	NA	NA	–	–	–	–	–	–	–	NA
CD34	–	NA	NA	NA	NA	NA	–	–	NA	NA	NA	NA	NA
CD68(KP-1)	–	NA	NA	NA	NA	NA	NA	NA	NA	NA	NA	NA	NA
E-Cad	–	NA	–	–	NA	–	–	–	NA	–	–	–	–
CycD1	N+	NA	NA	NA	N+	NA	NA	NA	NA	NA	NA	NA	NA
Inhibin-a	–	NA	–	–	–	–	–	–	–	–	–	NA	–
CK-P	FW+	NA	P+	–	NA	–	–	P+	F+	–	–	–	–
PR	–	NA	–	N+	–	F+	–	F+	–	–	–	–	–
WT-1	–	NA	P+	NA	NA	NA	NC+	NA	–	NA	NA	NA	NA
TG	–	NA	NA	NA	NA	NA	NA	–	–	NA	–	NA	NA
TTF-1	–	NA	NA	NA	NA	NA	NA	NA	NA	NA	NA	NA	–
S-100	–	NA	–	F+	NC+	NC+	–	NA	–	NA	–	NA	NA
Calretnin	–	NA	–	–	NA	NA	–	NA	–	NA	–	–	–
SMA	–	NA	NA	NA	NA	NA	NA	NA	NA	NA	NA	NA	NA
Desmin	–	NA	NA	NA	NA	NA	NA	NA	NA	NA	NA	NA	NA
Melan-A	–	NA	CW+	NA	NA	NA	NA	NA	NA	NA	NA	NA	NA
HMB45	–	NA	–	–	NA	NA	NA	NA	–	NA	–	NA	NA
CD117	–	NA	NA	NA	NA	NA	NA	NA	NA	NA	FM+	FM+	–
AFP	NA	NA	NA	NA	NA	NA	NA	NA	NA	NA	NA	–	–
PLAP	NA	NA	NA	NA	NA	NA	NA	NA	NA	NA	NA	NA	NA
CDX2	NA	NA	NA	NA	NA	NA	NA	NA	NA	NA	NA	NA	–
CEA	–	NA	NA	NA	NA	NA	NA	NA	NA	NA	NA	–	NA
EMA	–	NA	NA	NA	NA	NA	NA	NA	NA	NA	NA	NA	NA
GFAP	–	NA	NA	NA	NA	NA	NA	NA	NA	NA	NA	–	NA
Ki-67	3%	NA	1%-5%	1%-2%	NA	15%	NA	5%-10%	20%-30%	NA	NA	NA	NA
PAS stain	–	NA	NA	NA	NA	NA	+	NA	NA	NA	NA	NA	NA

Syn, synaptophysin; CgA, chromogranin A; CK-P, pan-cytokeratin; PR, progesterone receptor; TG, thyroglobulin; SMA, smooth muscle actin; AFP, alphafetoprotein; PLAP, placental alkaline phosphatase; CEA, carcinoembryonic antigen; EMA, epithelial membrane antigen; GFAP, glial fibrillary acidic protein; PAS stain, Periodic Acid-Schiff stain; +, positive; -, negative; N, nuclear; NC, nuclear and cytoplasmic; M, membrane; D, diffuse; F, focal; P, punctate; W, weak; FW, focal weak; DW, diffuse weak; MC, membrane and cytoplasmic; FM, focal membrane; DL, dot-like; NA, not available.

Imaging examinations play an important role in the initial diagnosis of SPN, notably utilizing CT and MRI modalities (14, 15). CT imaging typically reveals a predominantly cystic composition of the neoplasm, with distinct variations in the distribution of solid components. These solid components may manifest as papillary projections, attached nodules, or irregular slightly hyperdense areas floating within the cystic structure, forming the characteristic “floating cloud sign” indicative of SPNs. On the other hand, MRI findings often depict the neoplasm as a cystic solid lesion exhibiting a mix of T1 signal intensities and slightly elevated T2 signal intensities. Frequently, there is a lack of clear demarcation within the lesion, often accompanied by areas of hemorrhage, with constrained diffusion observed (16).

The morphological characteristics of primary ovarian SPNs closely resemble those observed in the pancreas (17, 18). Macroscopically, the neoplasm presents as a combination of cystic and solid components. Microscopically, the predominant growth patterns of the neoplastic cells include solid and pseudopapillary structures. Additionally, nest and microcystic formations can be identified, with the capsule typically containing gelatinous material. The neoplastic cells are typically solitary, featuring round or oval nuclei and lightly stained cytoplasm, occasionally exhibiting longitudinal nuclear grooves. In certain instances, prominent cytoplasmic vacuoles and eosinophilic bodies are evident. Notably, atypical or mitotic figures are notably absent. Within the solid region of the neoplasm, neoplastic cells may exhibit small foam-like cytoplasm or contain cholesterol crystals encircled by foreign body giant cells, resembling the frothy cells observed in the “cholesteroma-like” formations in the present case. To date, only five articles have referenced this distinctive structure. Further investigation is warranted to ascertain whether the “cholesteroma-like” formations represents a unique morphological feature of ovarian SPNs.

In immunohistochemical staining, β-catenin serves as the distinctive marker for SPNs (8). This unique expression is linked to the presence of gene mutations in β-catenin (CTNNB1), a critical component of the Wnt signaling pathway. The vast majority of SPNs exhibit CTNNB1 mutations, predominantly affecting exon 3 of the oncogene CTNNB1. These missense mutations impede the ubiquitination and subsequent degradation of β-catenin by proteasomes, leading to aberrant accumulation of β-catenin in the nucleus and cytoplasm. This dysregulation activates the Wnt/β-catenin signaling pathway, contributing to the pathogenesis of SPNs (19). In routine pathological practice, the abnormal accumulation of β-catenin is typically identified through immunohistochemical staining. Studies have reported the presence of two distinct CTNNB1 mutations (S37Y and p.S33C) in SPN (12). Additionally, CD99 paranuclear punctate positivity (20), while progesterone receptor (PR) consistently shows positive staining; however, E-cadherin, chromogranin A (CgA), alpha-inhibin, and calretinin are negative in SPNs. Despite the predominantly low-grade nature of SPNs, not all cases exhibit cytokeratin expression (12). In our case, focal cytokeratin expression (CK-P) was observed. Recent investigations have proposed ABCD1 as a novel diagnostic marker for solid pseudopapillary neoplasm of the pancreas (21).

The origins of SPNs remain a topic of debate (4). Earlier studies suggest an association between SPNs and hormone-responsive tissues, particularly those of the gonads. It is noteworthy that a significant proportion of pancreatic neoplasms are identified in females, with a frequent expression of progesterone receptors in these neoplasms (5). During embryonic development, there is a close developmental association between the ovarian reproductive ridge and the pancreas, suggesting the potential incorporation of primitive ovarian cells into the embryonic pancreas (22). This observation leads to speculation that SPNs may originate from cells of the ovarian reproductive ridge or primordial cells that were previously in proximity to the pancreas during early embryological stages (5).

Studies have documented the existence of ectopic pancreatic tissue in SPNs located in the mesentery, omentum, and colonic ovary (23, 24). Additionally, it has been suggested that pancreatic tissue may infiltrate ovarian dermoid cysts through secondary implantation. Ectopic pancreatic tissue has been identified in the liver as well, likely due to the shared embryological derivation of these from the primitive duodenum (25). In the absence of ectopic pancreatic tissue, the ovary represents the most frequent site of SPNs occurrence outside the pancreas. Moreover, the presence of pancreatic tissue in mature cystic teratomas is uncommon, with a reported incidence of only 1% as documented in Blackwell et al.’s classic study (26). In the present case, there is no indication of teratoma presence. Subsequent sampling and examination revealed the absence of ectopic pancreatic tissue or teratoma elements. MRI examination confirmed the lack of lesions in the pancreas. It is postulated that the neoplasm may have originated from germ-vestige or ovarian primordial cells that were in close proximity to the pancreas during early embryonic development.

Sex cord-stromal tumors typically exhibit positive E-Cad expression, SPNs are E-Cad negative (27). The cell regions with low cytoplasm and deep nuclear staining in SPN need to be distinguished from neuroendocrine tumors, which usually express epithelial and neuroendocrine markers, unlike SPNs (28, 29). In this case, the “cholesteroma-like” formations is similar to steroid cell tumors; however, it lacks expression of inhibitors, calretinin, and Melan-A (30). This is currently the only case of SPN with a cholesterol tumor like structure as an important component. Ovarian epithelial derived cancers usually exhibit significant cellular atypia, express epithelial markers, and exhibit a high proliferation index, while the epithelial like cells in SPN display subtle morphology and extremely low proliferation index (31–34). Both yolk sac tumors and SPN show solid epithelioid and papillary structures. However, yolk sac tumors also exhibit microcystic and endodermal sinus like structures, and express AFP, SALL4. It is important to differentiate the papillary region of SPNs from malignant ovarian goiter (papillary thyroid carcinoma) (35). The nucleus of papillary carcinoma is ground glass like, with visible nuclear grooves, and immunohistochemical expression of TG and TTF-1.

Various tumors harbor mutations in the CTNNB1 gene, including microcystic stromal tumors of the ovary (MST), soft tissue cribriform fibromatosis, juvenile nasopharyngeal angiofibroma, and sclerosing pulmonary hemangioma (36). While the precise mechanism remains uncertain, MST may involve ovarian stromal cells, with CTNNB1 point mutations leading to activation of the WNT/β-catenin pathway. This activation pathway mirrors the mechanisms observed in solid pseudopapillary neoplasms of the ovary (37). Generally, the tumor presents as predominantly solid with occasional cystic or hemorrhagic areas, imparting a spongy appearance. Histologically, it consists of three main components: the microencapsulated region, the solid cell region, and the fibrous stroma. Cells within the solid region are round or oval, akin to those seen in the solid region of ovarian SPNs. However, microencapsulated structures, a hallmark of MST, are typically absent in SPNs, and pseudopapillary structures are not a feature of MST (38). Immunohistochemically, MST demonstrates positive staining for WT-1, FOXL2, and SF-1 (39), whereas SPN exhibits no expression, providing a differentiating characteristic.

Based on data, the metastasis rate of primary ovarian SPNs is approximately 15.38%. In a case documented by Syriac, metastasis was identified in the fallopian tubes and spread to the liver, small intestine, perirectal area, perilesional left ureter, omentum, abdominal wall, and vagina. Tragically, the patient succumbed to the disease 8 months following the initial diagnosis (6). As documented in the case by Kushner, recurrence occurred after 6 months with subsequent metastasis to lymph nodes (13). The primary treatment approach for ovarian SPNs typically involves surgical intervention, complemented by radiotherapy and chemotherapy (3). Furthermore, the potential therapeutic targeting of the oncogene CTNNB1 in diverse diseases warrants further exploration to determine if molecular alterations can be managed uniformly across various conditions.

## Conclusion

This case report presents a solid pseudopapillary tumor originating from the ovary, notably distinguished by the presence of “cholesteroma-like” formations, a feature not previously documented in other SPNs. Further research is warranted to ascertain the uniqueness of this characteristic in ovarian SPNs.

## Data Availability

The original contributions presented in the study are included in the article/Supplementary Material. Further inquiries can be directed to the corresponding author.
